# A cognitive brain–computer interface monitoring sustained attentional variations during a continuous task

**DOI:** 10.1007/s11571-019-09521-4

**Published:** 2019-02-08

**Authors:** Antoine Gaume, Gérard Dreyfus, François-Benoît Vialatte

**Affiliations:** 1 ESPCI Paris, PSL Université Paris, Paris, France; 2 CNRS UMR 8249, Brain Plasticity Unit, Brain-Computer Interface Team, Paris, France; 3 EPF École d’ingénieur, Sceaux, France

**Keywords:** Brain–computer interface, EEG, Attention, CPT, Cognitive BCI

## Abstract

We introduce a cognitive brain–computer interface based on a continuous performance task for the monitoring of variations of visual sustained attention, i.e. the self-directed maintenance of cognitive focus in non-arousing conditions while possibly ignoring distractors and avoiding mind wandering. We introduce a visual sustained attention continuous performance task with three levels of task difficulty. Pairwise discrimination of these task difficulties from electroencephalographic features was performed using a leave-one-subject-out cross validation approach. Features were selected using the orthogonal forward regression supervised feature selection method. Cognitive load was best predicted using a combination of prefrontal theta power, broad spatial range gamma power, fronto-central beta power, and fronto-central alpha power. Generalization performance estimates for pairwise classification of task difficulty using these features reached 75% for 5 s epochs, and 85% for 30 s epochs.

## Introduction

Since its first definition by William James a century ago (James 1890), attention has been generally defined by its cognitive role in the information processing occurring in the brain, rather than by its neuronal substrate or physiological function. This way of defining attention is what makes it so complex and difficult to study (Richard 1980).

The first cognitive models of attention describe it in terms of filters (Treisman 1964; Deutsch and Deutsch 1963; Norman 1968). In these models, percepts are filtered according to their relevance to the organism to be driven towards working memory for conscious processing and possibly long-term storage. Afterwards, the notion of attentional load emerged: attention was modeled as a limited capacity system (Kahneman 1973), where limited mental resources are shared between the different information-processing tasks the brain has to run. Attention becomes responsible not only for selection of relevant activities, but also for inhibition of distracting stimuli. This model includes every cognitive task the brain has to run in his theory of attention and not simply the process of selecting between competing simultaneous stimuli, on which the earlier bottleneck theories had focused. The attentional capacity of a person can vary depending on his arousal (Berlyne 1960), which, in turn, can be influenced by the attended tasks—this is for instance one explanation of driver’s drowsiness (Chen et al. 2018; Zeng et al. 2018). Later on, attention models became modular and more executive (Johnston and Heinz 1978; Posner and Snyder 1975): attention is considered as a flexible mental process, where voluntary control of attention requires more resources than automatic attentional discrimination. Attention was finally modelled from a neurocognitive perspective (Posner and Petersen 1989; Petersen and Posner 2012), as a unified system for the control of mental processing involving a network of anatomical areas independent from the sensory input processing areas of the brain, with three main subsystems of attention: *alerting*, *orienting* and *detecting*.

Attention has several submodalities [e.g. overt or covert attention, focused attention, selective attention, divided attention, alternating attention, vigilance, see Gaume et al. (2015) for a detailed discussion]. We are interested specifically in sustained attention, i.e. the self-directed maintenance of cognitive focus in non-arousing conditions while possibly ignoring distractors and avoiding mind wandering (Roberton and Garavan 2004; Clayton et al. 2015; Gaume et al. 2016). This state of maintaining attention is related to what is called concentration outside the field of neuroscience. Some authors use the term sustained vigilance or just vigilance to designate this state (e.g. Warm et al. 2008), but it is more widely accepted that vigilance refers to a condition of sustained *arousal* with the purpose of ensuring that important or surprising percepts will not be missed (Gaume 2015) (whereas we are interested in this study in the maintenance of overt attention towards a *non surprising* or even *boring* stimulus without mind wandering).

A recent framework for visual attention (Knudsen 2007) defined attention as a competitive selection process. This framework is well-compatible with real-time modelling in cognitive BCI. It introduces an explicit relationship between attentional networks and the direct control of the sensory organs that generate the incoming stimuli. It also integrates the concept of salience filters to describe the set of brain networks that automatically estimate the pertinence of an incoming sensory input (Gaume et al. 2016; Gaume 2015). The present investigation was grounded in Knudsen’s attentional model, with a neuro-cognitive perspective coming from EEG modeling, where attention is considered as the result of three coordinated processes (Stuss et al. 1995; Clayton et al. 2015):

(A) Monitoring and evaluation of ongoing cognitive processes, competitive selection process to determine which information gains access to working memory (Desimone and Duncan 1995), therefore linking working memory, attention, and task load.
(B) Excitation of task-relevant processes.
(C) Inhibition of task-irrelevant processes.

The cognitive control involved in sustained attention tasks is correlated with frontal midline theta-band activity (Cavanagh and Frank 2014; Clayton et al. 2015; Wokke et al. 2017), congruent with [A] monitoring processes. Increased gamma-range oscillations are believed to promote task-relevant activity (Reinhart et al. 2011; Akimoto et al. 2013; Ahveninen et al. 2013; Potes et al. 2014; Clayton et al. 2015) congruent with [B] excitation processes. Alpha power has been linked with inhibition of task-irrelevant sensory modalities in task-irrelevant cortical areas (Makeig and Inlow 1993; Bollimunta et al. 2008; Snyder and Foxe 2010; Toscani et al. 2010; Anderson and Ding 2011; Mazaheri et al. 2014; Clayton et al. 2015; Wang et al. 2016), congruent with [C] inhibition processes.

Brain–computer interfaces (BCIs) are communication systems that enable a direct and real-time exchange of information between the brain and the external world [see Nicolelis (2011) for an introduction to this subject]. The first BCI development attempt, which also served as a proof of concept, was carried out in 1973 by Jacques Vidal and his team in California. Their experiment was called the “BCI project” and *was meant to evaluate the feasibility and practicality of utilizing the brain signals in a man–computer dialogue* (Vidal 1973). The goal of BCI systems is to create communication pathways that differ from the normal input/output channels used by the brain, namely the sensory organs to capture information about the world and the peripheral nervous system coupled with the muscles to interact with the environment (Wolpaw et al. 2000). The purpose of such alternative pathways is frequently viewed as a means of assisting in the rehabilitation of disabled or paralysed persons, to whom BCIs can be of great help by either replacing a defective sensory input or providing substitute ways to interact with the world. However, many other applications can emerge from the development of real-time brain signals decoding and stimulation techniques. They include applications of neurofeedback, and applications outside of the medical world, such as in BCI games (Marshall et al. 2013), performance analysis (Tharawadeepimuk and Wongsawat 2017), sonification (Sayan et al. 2019), silent communication devices^1^ or ways to improve cognitive activity.^2^

Thorsten Zander introduced a slightly different classification in which exogenous BCIs are referred to as reactive while endogenous interfaces are separated into active and passive, respectively if the subject consciously triggers control signals or if the interface passively monitors the user’s brain state (Zander et al. 2010; Zander and Kothe 2011). To further refine this classification, we call sensory BCI a device with a control signal that is a correlate of sensory processing, motor BCI a device that uses activity from the motor cortex, and cognitive BCI a system that monitors cognitive functions (Gaume et al. 2015). The long-term goal of our research is the development of a cognitive BCI able to monitor the variations of sustained attention in real time. This “attentionometer” could for example warn its user immediately and objectively that its attention shifted towards a distractor. It could also be used to determine its user’s ability to pay attention at certain moments of the day, for example to know he is capable of driving. Such a device could also be used to train sustained attention by providing a continuous feedback to the user. More generally, being able to monitor our attention as directly as, for instance, the position of our arm, would probably allow us to learn how to consciously regulate our attention, and to find ways to concentrate easily and comfortably during long periods of time. This is the principle of neurofeedback (Lachaux 2011; Arns et al. 2017; Gaume et al. 2016).

The challenge that is adressed in the present work is to find a neural correlate of the variations of sustained attention that can be monitored in real time and that, ideally, does not require invasive hardware nor the performance of a specific task. Classical approaches to monitor sustained visual attention are based on the Mackworth Clock (Mackworth 1948), and involve long sessions of visual monitoring in search for unusual events. The objective measure used in that case as an indicator of attention is the response time of the subjects to the appearance of targets. However, changes in sustained attention can be measured either as fluctuations, or deteriorations of performances along time (Clayton et al. 2015). Most subjects indicate that their attention fluctuates between targets and that each unusual event recaptures their focus. The discrete response time measurements used in such experiments are therefore more related to deteriorations of attention, while the experiment fails to capture the fluctuations occuring in between the targets. In addition, all subjects who show a significant increase in their response time over the course of the experiment report drowsiness and dozing. Consequently, it is difficult to disambiguate such an experiment from the monitoring of alertness and vigilance (i.e. sustained arousal). Therefore, we deem this experimental approach not optimal for the monitoring of sustained attention. Indeed, it only requires a discrete involvement of attention and only allows a discrete measurement of performance. Consequently, the design of our cognitive BCI involves a continuous task: the subject has to focus continuously, the evaluation of attentional load relies on continuous stimulations, and the performance of the subject is measured continuously.

We therefore introduce an experimental paradigm involving a continuous task for the monitoring of variations of visual sustained attention. Furthermore, we intend to design a task that minimizes the involvement of cognitive functions other than sustained attention. We therefore decided to focus on the attention required to continuously update the visual information we get from our sensory inputs instead of a task based on continuous processing of the information stored in working memory, which would involve working memory load in addition to sustained attention.

## Materials and methods

### Continuous performance task (CPT)

Continuous Performance Task (CPT) is a test used in neuropsychology for the assessment of sustained and selective attention. Sustained attention is the ability to maintain concentration over time on a given task while selective attention refers to the ability to focus on relevant stimuli in a distracting environment. CPT paradigms generally involve multiple repetitions of a rapid presentation of stimuli with infrequently occurring targets. More details about CPTs can be found in Riccio et al. (2002). The task we developed is different from static CPT but keeps the idea that the evaluation of sustained attention requires a continuous involvement of the subject. Due to the bias-variance dilemma (Geman et al. 1992), if sources of variability exist in the data, it creates a lower bound (the so-called *irreducible error*) for classification accuracy. We therefore wanted to avoid discrete evaluations of performance, in order to constrain the subject to maintain continuously his attention without fluctuations. Indeed, such fluctuations would induce label noise in our database, which would increase the *irreducible error*.

Ecological stimuli are of great importance for BCI designs (Huang et al. 2018). *On the move* estimation of attentional mechanisms (continuous estimation) using video games is more ecological than classical static displays (discrete estimation) (Szalma et al. 2014). In our experiment, the subjects play a driving video game using a joystick: they have to maintain overt visuo-motor cognitive control, involving both the maintenance of cognitive control and visual attention [see e.g. Anguera et al. (2013) about driving video games and attention). It also involves mechanisms unrelated with sustained attention itself, such as arousal, stress regulation, sensory integration of visual cues, and the motor planning and execution of the task. However, these unrelated mechanisms are present in both the low, medium and high difficulty conditions. Our task design was aimed at increasing attention-related functional loads differentially according to task difficulty. In other words, despite the task itself is not measuring only sustained visual attention, the variation of difficulty is more specific to it. This attentional engagement was confirmed by two observations: first, subjects involved in higher difficulties reduced their eye blink frequency, a known correlate of visual attention [see e.g. (Kim et al. 2014)]. Second, they lost the ability to answer direct verbal questions in the hard condition [attentional inhibition typical of *attentional tunneling* (Wickens and Alexander 2009)].

**Fig. 1:**
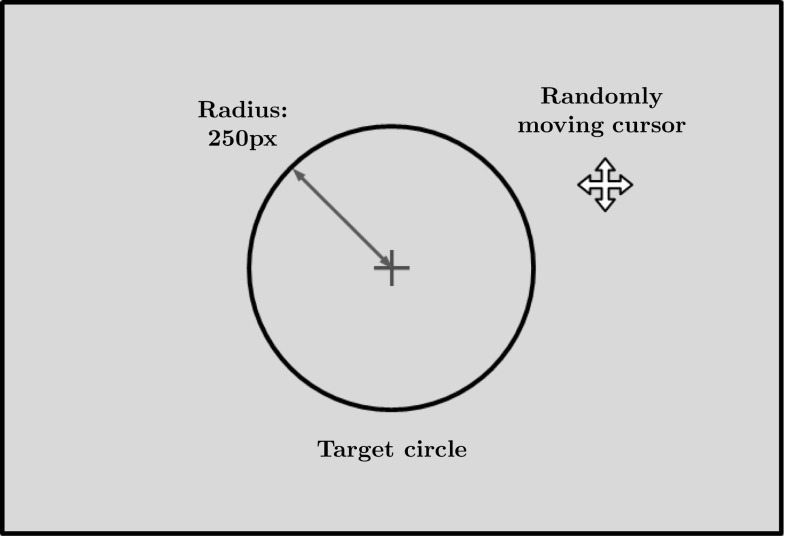
Illustration of the CPT interface. The subject of the experiment tries to keep a randomly moving cursor inside the target circle using a joystick. The difficulty of the task can be adjusted by changing the speed of the cursor

Our task consists in the motor control of a cursor using a joystick. The concept is simple: subjects of the experiment sit in front of a computer screen displaying a black circle (the target) on a grey background (see Fig. 1 for an illustration). A cursor moves randomly and continuously on the screen and subjects are asked to keep this cursor inside the circle using the right joystick of a joypad (EG-C1036, Targetever Technology Co. Ltd.). The difficulty of the task is adjusted by modifying the speed of the random movement. Performance of the subjects can be monitored in real time based on the correction applied to the random movement. The whole experiment was developed using PsychToolBox-3 for MATLAB (Brainard 1997; Kleiner et al. 2007) and displayed on a 120 Hz screen with a resolution of $1920 \times 1080$ pixels.

### Experimental procedure

After installation of the electrodes and presentation of the EEG principles and signals, subjects were trained on the CPT several times at low speed (150 px/s) to make sure they understood how to manipulate the joypad.

A calibration session was then performed, during which each subject played the game at 20 different difficulty levels (starting at 75 px/s and up to 1500 px/s, increasing the speed by 75 px/s at the beginning of each sequence). Each level was played continuously for 20 s and subjects controlled when to begin the rounds so they could take breaks in-between. Data from the calibration phase was used to determine the speed of the pointer during the rest of the experiment. This calibration phase lasted less than ten minutes.

The recording session then started. Each subject played a total of 60 rounds of the game at three different difficulty levels (20 “easy”, 20 “medium” and 20 “hard”). The first round was an “easy” round, followed by a “medium” round and a “hard” round. This was repeated 20 times, in other words the presentation order was *easy-medium-hard-easy-medium-hard-easy-medium-hard*-etc. Each round lasted 30 s for a total duration of around 40 minutes. The subjects controlled when to begin the rounds so they could take breaks in-between. The cursor speed for the “easy” levels was always 150 px/s. Cursor speeds for “medium” and “hard” levels were determined according to calibration results as the speeds for which the cursor would stay 95% and 50% of the time in the circle respectively. Speed ranges were [375, 750] px/s for “medium” level and [650, 900] px/s for “hard” levels, depending on the subject. Last round score, best scores and average scores (percentage of time spent by the cursor inside the circle) for each difficulty were shown to the subject between each round to stimulate his/her motivation.

### Experimental conditions

EEG recordings took place in a dark room, where subjects were seated in a comfortable armchair, about one meter away from the screen used to display the CPT. The subjects were shown their EEG activity prior to the recording and explanations were given about muscular artefacts and eye blinks. They were instructed to relax and prevent excessive muscular contractions or eye movements.

### Subjects

Seventeen (17) healthy subjects took part in the experiment. Three (3) of them were rejected from the study because the recorded data were too noisy.^3^ Fourteen (14) subjects remained, among which eleven (11) were males and three (3) females, with an average age of 23.7 (SD 3.9, range 19–32). All had normal or corrected-to-normal vision and none of them had any known history of epilepsy, migraine or any other neurological condition. The study followed the principles outlined in the Declaration of Helsinki. All participants were given explanations about the nature of the experiment and signed an informed consent form before the experiment started.

### Data acquisition

**Fig. 2:**
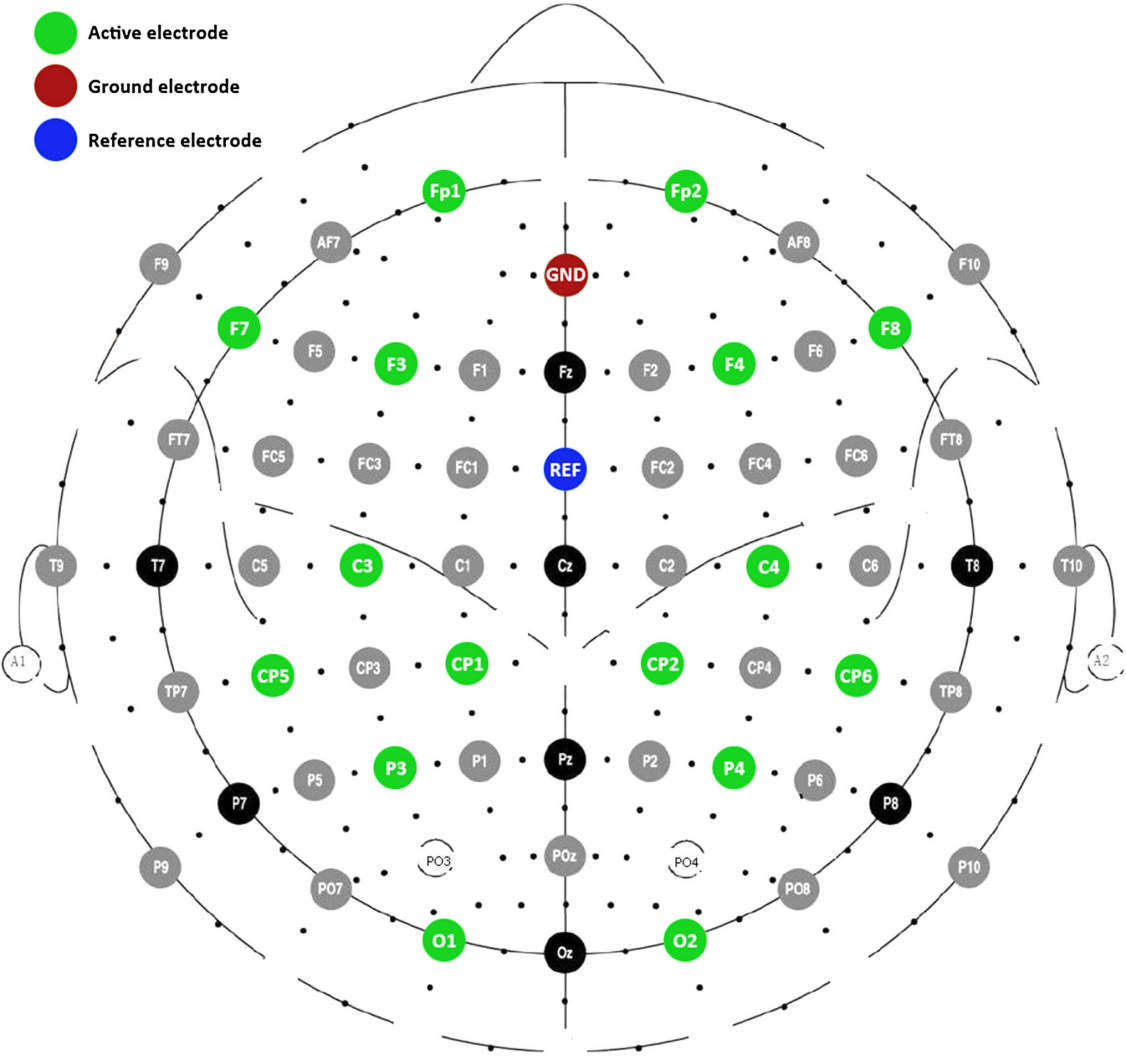
Electrode placement for CPT recordings. Brain activity was recorded using 16 active electrodes (in green), located all over the scalp with a focus on the frontal, parietal and occipital regions which cover several regions involved in attention and visual processing. (Color figure online)

EEG signals were continuously recorded at a sampling rate of 2 kHz using 16 active Ag/AgCl electrodes from an actiCap system, connected to a V-Amp amplifier, both from Brain Products. The electrodes were placed according to the 10–20 system with a focus on frontal, parietal and occipital regions at positions Fp1, Fp2, F7, F3, F4, F8, C3, C4, CP5, CP1, CP2, CP6, P3, P4, O1 and O2, as illustrated in Fig. 2. Two additional electrodes were used as ground and reference and were located respectively at AFz and FCz.

### Signal processing

Analyses were performed using MATLAB 2013a. The recorded EEG signals were filtered between 0.5 and 90 Hz using a zero-phase 3rd-order digital Butterworth filter, and the same kind of filter was applied around 50 Hz to remove the power-line noise. Filtering was applied on the raw signals before any segmentation to avoid boundary effects.

### Eye blink rejection

Preliminary experiments showed that subjects produced fewer eye blinks when they were engaged in tasks requiring a high level of visual attention than in other tasks. In order to avoid classification of our data based on the number of eye blink artifacts found in EEG signals, we needed either to remove all epochs containing eye blinks, thereby reducing significantly the size of our dataset, or to find a way to remove eye blinks from EEG signals.

Therefore, a Second-Order Blind Identification (SOBI) algorithm from the EEGLAB toolbox was used to decompose recorded EEG signals into independent components. Eye blink activity and strong eye movements artefacts were removed before signal reconstruction from SOBI components. Details about this algorithm can be found in Belouchrani et al. (1993). More information about the eye blink removal using ICA can be found in Jung et al. (2000).

### Feature extraction and selection

This section presents results obtained using only spectral features extracted from EEG power spectra. All features were extracted from epochs of 1, 3, 5, 10 and 30 s. Absolute and relative EEG power in the delta ($\delta$ 1–4 Hz), theta ($\theta$ 4–8 Hz), alpha ($\alpha$ 8–12 Hz), low beta ($\beta -$ 12–18 Hz), high beta ($\beta +$ 18–25 Hz), low gamma ($\gamma -$ 25–35 Hz) and high gamma ($\gamma +$ 35–45 Hz) frequency bands were extracted from each channel of each EEG epoch, accounting for a total of 224 features per epoch (14 features and 16 channels). Absolute power refers to the total power in a given frequency band, obtained using FFT and a Hanning window on a given epoch. Relative power refers to the ratio of the absolute power in a given frequency band to the absolute power in the whole spectrum, taken between 1 and 45 Hz.

We used analysis time windows of 1, 3, 5, 10 and 30 s. These epoch lengths span in an exponential progression the [1–30] s range, so we could test more short windows than large windows. We tested these different time windows in order: (1) to test for stability to parameter changes, and (2) to provide a global overview of the tradeoff between accuracy and speed.

When classification was not based on a single variable, candidate variables were ranked in order of decreasing relevance by Orthogonal Forward Regression (OFR); feature selection was performed by the random probe method, as described in Stoppiglia et al. (2003). Random variables (probes) were added to the feature set and only variables that ranked better than 95% of the probes were kept for classification (35 features out of 224).

### Classification

Classification of the EEG epochs was performed using Linear Discriminant Analysis [LDA, see for instance Fukanaga (1990)]. LDA estimates separating hyperplanes in the feature space by seeking the direction, in feature space, such that the projections of the classes on this direction have maximum iner-means distance and minimum variance. Likelihood ratios based on the distance to these hyperplanes are used to assign new observations to the different classes. LDA has the advantages of a very low computational requirement, and a single solution with a given training set. This classifier is simple to use, and has been used with success in a great number of BCI (Lotte et al. 2007). LDA assumes Gaussian distributions, which holds true for our features.

Epochs were labelled as “easy”, “medium” or “hard”, depending on the difficulty of the task. We performed pairwise classification (Knerr et al. 1990) (between all pairs of classes) and three-class classification. Since the datasets used for classification were balanced, expected classification accuracies using random features were 50% for pairwise classification and 33.3% for three-class classification. Feature selection and classifier training were performed using data from all subjects except one and data from the remaining subject were used for validation. This is repeated for each subject and accuracies are averaged (Leave-One-Subject-Out, LOSO).

## Results

### Single-feature classification

Table 1 shows the evolution of the best accuracy obtained using a Bayes classifier (i.e. a single feature LDA) as a function of the epoch length. For each discrimination, we selected the feature that ranked first in the ranking provided by the OFR method. It can be observed that the best accuracy increases with epoch length for all classification scenarios. However, 30 s epochs seem to be only slightly easier to classify than 1s epochs using a single spectral power feature.

**Table 1:** Best accuracies using a single spectral power feature for different epoch lengths and for the four classification scenarios described in “Classification” section

Epoch duration (s)	3-Class classifier (%)	“Easy” versus “medium” (%)	“Easy” versus “hard” (%)	“Medium” versus “hard” (%)
*Best LOSO classification accuracies*				
1	40.5	60.1	60.8	54.5
3	42.2	62.7	63.1	55.8
5	42.8	64.3	65.0	57.1
10	44.0	65.5	65.8	58.2
30	46.2	68.8	66.4	60.2

**Table 2:**
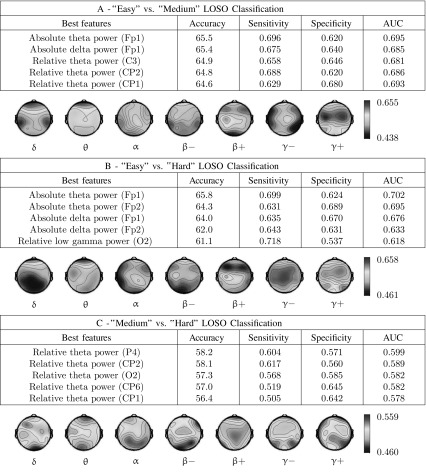
Two-class classification results obtained using a single spectral power feature when separating EEG epochs of 10 s recorded during two discriminations among “easy”, “medium” and “hard”

The five features giving the best average accuracies are listed for each classifier. Sensitivities and specificities are given for the best threshold. Accuracies obtained using absolute EEG power for each frequency band and each channel are presented as topographic maps (frontal electrodes are located at the top). Details about frequency bands can be found in “Feature extraction and selection” section

Tables 2 and 3 illustrate the results obtained on 10 s epochs using single feature LDA for respectively three-class classification (“easy” vs. “medium” vs. “hard”) or pairwise classification (between any pair of difficulty levels). We observe that the best accuracies obtained in “easy” versus “medium” (65.5%) and “easy” versus “hard” (65.8%) discriminations come from the same feature (absolute theta power at Fp1) and are significantly higher than the accuracies obtained for “medium” versus “hard” classification. The accuracy maps obtained with absolute spectral power are also very similar in the “easy” versus “medium” and “easy” versus “hard” discriminations, and both qualitatively look like the accuracy map obtained in the 3-class discrimination. We also observe that the features giving the best results are mostly low frequency features (delta and theta band power) in the prefrontal, superior parietal and central cortices. These observations are similar with other window lengths (not illustrated).

**Table 3:**
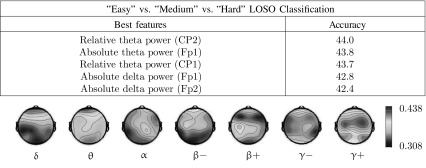
Three-class classification results obtained using a single spectral power feature when separating EEG epochs of 10 s recorded during “easy”, “medium” and “hard” discriminations

The five features giving the best average accuracies are listed. Accuracies obtained using absolute EEG power for each frequency band and each channel are presented as topographic maps (frontal electrodes are located at the top). Details about frequency bands can be found in “Feature extraction and selection” section

### Multiple-feature classification

Tables 4 and 5 show the results obtained using LDA classification with multiple features in the four discriminations described in “Classification” section. All results are given for different epoch lengths ranging from 1 to 30 s. Accuracies are obtained using a leave-one-subject-out cross validation method. Along with the classification accuracies, the best features selected by OFR for the classification of 10 s epochs are listed for each discrimination.

**Table 4:**
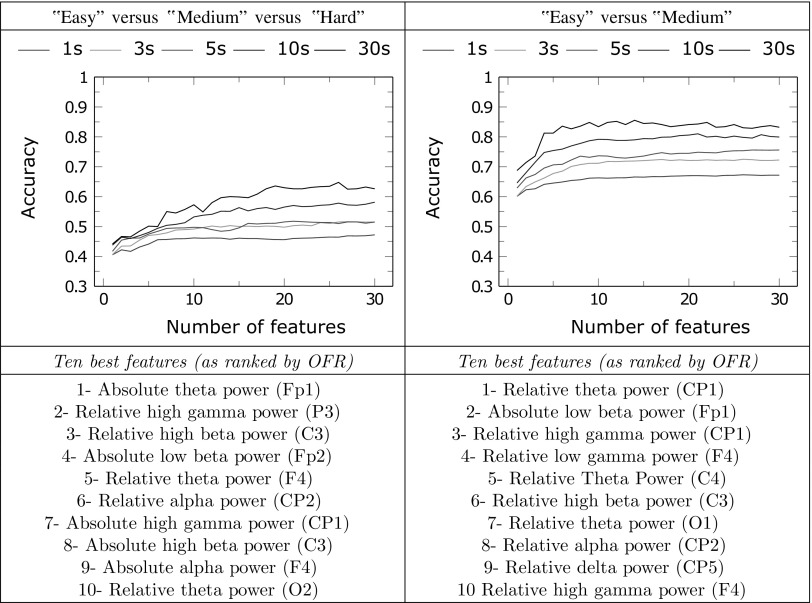
Classification results using multiple features for three-class classification (left) and “easy” versus “medium” classification (right)

Accuracies are given for different epoch lengths as a function of the number of features used by the classifier. The best ten features selected by OFR on 10 s epochs are listed for both classifiers

**Table 5:**
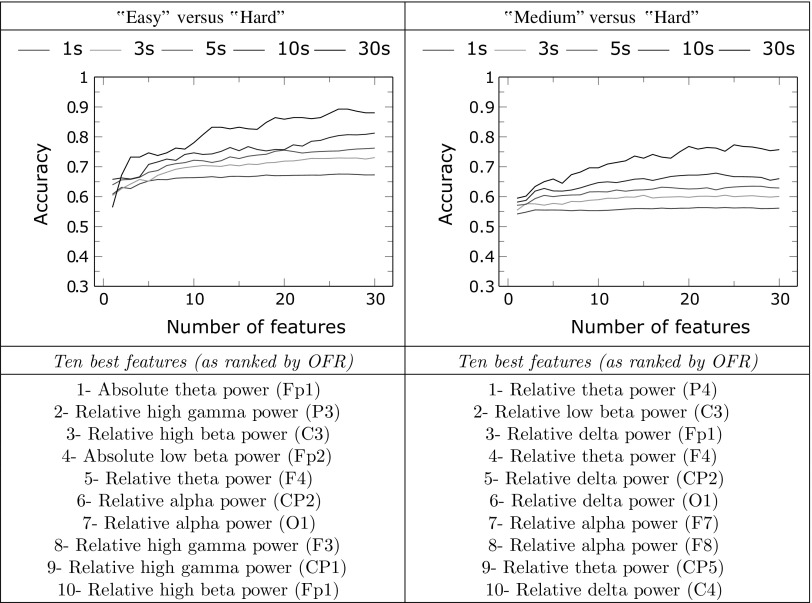
Classification results using multiple features for “easy” versus “hard” classification (left) and “medium” versus “hard” classification (right)

Accuracies are given for different epoch lengths as a function of the number of features used by the classifier. The best ten features selected by OFR on 10 s epochs are listed for both classifiers

On average, LOSO classification accuracies increase with the number of features for all discriminations and all epoch lengths. Using 30 s epochs, accuracy reaches 64.8% for the three-class classifier using 26 features; 85.5% in the “easy” versus “medium” discrimination using 14 features; 89.3% in the “easy” versus “hard” discrimination using 26 features and 77.3% in the “medium” versus “hard” discrimination using 25 features. With shorter 5 s epochs, a situation more likely to happen in a real cognitive BCI, accuracy reach 51.8% with the three-class classifier; 75.6% in the “easy” versus “medium” discrimination; 76.2% in the “easy” versus “hard” discrimination and 63.5% in the “medium” versus “hard” discrimination.

It can be noted that the “easy” versus “medium” classification is the discrimination that reaches its accuracy plateau with the lowest number of features, thereby decreasing the risk of overfitting.

The features selected for each classifier are different and, as expected, not directly linked with the features giving the best accuracy when classification is performed using a single input. However, except for the “medium” versus “hard” classifier, which uses several features in the delta range (1–4 Hz), the other three classifiers select similar features among their best ten including:

- Several features based on absolute or relative theta power (4–8 Hz), taken from multiple locations over the scalp, from frontal to occipital regions,
- features based on high gamma power (35–45 Hz), taken in the central or parietal regions,
- a feature based on frontal low beta power (12–18 Hz),
- a feature based on central high beta power (18–25 Hz),
- a feature based on centro-parietal alpha power (8–12 Hz).

### Subjective feedback

After the experiment, each subject was asked about his perception of the difficulty levels of the game.

- All subjects found the “easy” rounds very easy. Some reported boredom. On average, 99.8% of “easy” play time was spent inside the target circle.
- Most subjects reported that the “medium” levels were the most interesting and engaging, as they had the impression of having a real control over the movement of the cursor. 94.6% of total “medium” play time was spent inside the target.
- All subjects found that “hard” levels were by far the hardest, and most of them reported that it was slightly less motivating than the “medium” difficulty because they felt they did not have enough control over the fast-moving cursor. Some subjects however found this difficulty very challenging and interesting. On average, 56.4% of “hard” play time was spent inside the target circle.

In order to control that the task had an effect on cognitive load, we confirmed on three subjects the effect of variations in task difficulty, using the NASA Task Load Index (Hart and Staveland 1988). The six dimensions (Mental Demand, Physical Demand, Temporal Demand, Performance, Effort, and Frustration) of the scale were compared between “easy” versus “medium”, as well as “medium” versus “hard” conditions, using a Wilcoxon paired test with Bonferroni corrections for multiple comparisons. Task difficulty change led to significant increase of Mental Demand ($p < 0.05$ in all subjects and both conditions), there were no significant changes with the other dimensions of the scale ($p > 0.10$ in all subjects and both conditions).

## Discussion

Subjects of the experiment reported that the “medium” rounds and the “hard” rounds required more or less the same concentration. We therefore expected that classification would work better between “easy” and “medium” or between “easy” and “hard” rounds, than between “medium” and “hard” rounds. This is consistent with the results shown on the tables. It is also consistent with the fact that the features selected to discriminate “medium” and “hard” rounds are different from those selected for the other classifiers.

In order to estimate BCI performances, LOSO classification accuracies were used. Classical BCI models are designed and tested within subjects. Instead, LOSO scores are estimates of the generalization performance of the classifier onto new subjects, and it is therefore an estimate of transfer learning (Cheng et al. 2017). The choice of the LOSO validation approach was made to avoid biases in the estimates. Indeed, we might achieve higher accuracies on this database using within-subject analysis instead of cross-subject classifications. However, a within-subject model would increase the risk of modeling subject-related motor and/or cognitive confounders, which would be a serious flaw for a cognitive BCI (Gerjets et al. 2014). Furthermore, a LOSO approach allows us to design a cognitive BCI system which does not need calibration. In other words, it can be applied right away on new subjects without retraining.

Pairwise classification (“easy” vs. “medium” or “hard” rounds) reached 75% even with short 5 s epochs. Accuracies above 85% were obtained using 30 s epochs. Note that these accuracies were obtained without any calibration of the model to the left-out subject. Due to the bias-variance dilemma (Geman et al. 1992), if sources of variability exist in the data, it creates a lower bound (the so-called *irreducible error*) for classification accuracy. Considering that BCI illiteracy affects 15–30% of the population (Jeunet et al. 2016), the irreducible error is 15%, and therefore accuracies higher than 85% would be suspicious and might indicate a flawed model. These accuracies are therefore close to the optimum, which illustrates the robustness of the BCI model and show that prediction of the current cognitive load required by a task can be monitored using EEG. Such performances are largely sufficient for several biomedical applications. Indeed, neurofeedback and monitoring applications do not need a high accuracy, but a high specificity (above 85%), so that it would trigger only useful alarms. We can achieve such a detection by increasing specificity at the cost of sensitivity, consequently our system can already be used for biomedical applications in its present state.

In the “easy” versus “medium” discrimination, the LOSO classification accuracy estimations reached a plateau when the number of features exceeded ten, thereby proving that the LDA classifiers designed with these features are not very prone to overfit our data. However, in other discriminations, and especially in the “easy” versus “hard” discrimination, accuracy increased with the number of features and did not seem to reach a plateau (see Table 5).

Frontal theta power was a good predictor of the task load, providing a classification accuracy of 66% to separate the “easy” task from higher demand “medium” and “hard” tasks. This result is in accordance with previously reported observations: an increase of frontomedial theta is the best known correlate of sustained attention (Klimesch 1999; Clayton et al. 2015). Joint EEG and fMRI analysis have shown that prefrontal theta oscillations play a key role in salience and executive functions (Mas-Herrero and Marco-Pallars 2016) serving as a common substrate for the engagement of pre-supplementary motor area and the dorsomedial prefrontal cortex. Knudsen’s model does consider salience filters and executive functions as core components of attention (Knudsen 2007). Augmentation of frontal theta has also been widely reported in association with working memory load (Gevins et al. 1998; Jensen and Tesche 2002; Gevins and Smith 2003; Sauseng et al. 2005; Cavanagh and Frank 2014; Hsieh and Ranganath 2014; Fairclough and Ewing 2017).

High beta activity in the left central region provided good classification results. This activity may be linked with motor control, since the task involved the control of a joystick with the right hand. Nevertheless, the high beta frequency band, containing spectral activity between 18 and 25 Hz, does not overlap with the sensorimotor rhythm, and may therefore be linked with cognitive load and attention more than with direct control of motor activity. This is consistent with the work of Wróbel (2000), which supports that the 15–25 Hz frequency band is a general carrier for attention in the brain. Hypotheses about the sources of this frontal activity could be made, however precise reconstruction would require a high number of electrodes (64$+$), which is why this method has not been used with our equipment.

Attention is based on brain networks involving coupling of several frequencies (Clayton et al. 2015). In order to target these complex interactions, the feature selection approach which was used in our investigations extracts biomarkers carrying complementary information. Instead of relying on one single marker, the resulting cognitive BCI model combines multiple features (see Table 4). Overall, the attentional load is predicted by a combination of prefrontal theta power, broad spatial range gamma power, fronto-central alpha power and fronto-central beta power. This combination of channel and frequency ranges is coherent with the existing knowledge about the underlying networks of attention: frontomedial theta rythms for attentional monitoring processes, gamma-range oscillations for excitation processes, and alpha power for attentional inhibition processes. As we already explained above, the beta range activity could be correlated with either cognitive load or sensorimotor confounders of the task.

Another potential confounder is the variation of arousal during the task. The session was organized with successive trials of increasing difficulty, which may induce a correlation of mental fatigue with the task condition (the very first trial was always easy, the very last always hard). A randomized order would be a better control for this mental fatigue confounder. However, a randomized order would also introduce an anxiety confounder : not knowing the level of difficulty in advance was more stressful when the difficult condition occurred, whereas anxiety levels were more comparable between conditions when the subjects could anticipate it. Both mental fatigue and anxiety can have an impact on arousal, we chose to prevent anxiety.

## Conclusion

To conclude, we presented in this paper an experiment aimed at discriminating low versus high sustained attention states in a continuous task. This continuous task approach introduces a new type of cognitive BCI design, where we attempt to control the cognitive load using an ecological video-game where a cognitive constraint is continuously maintained to prevent load fluctuations. We showed that features such as average frequency band power, which can be estimated continuously and do not require discrete stimulations, provide good classification rates even with short epochs (5 s). A leave-one-subject-out cross-validation approach was used to avoid overfitting. Nevertheless the present investigation is yet insufficient to prevent modeling confounders of attentional load. For instance, event though we removed eye blinks using SOBI, we cannot rule out that some features participating in the classification process may be related to eye movement or electromyographic artefacts, which might act as a potential confounder of sustained attention. Consequently, other experiments will be required to differentiate potential confounders (Gerjets et al. 2014), i.e. the features specific to the motor control task of this investigation, emotional, arousal or stress correlates [see for instance Mühl et al. (2014)], artifacts, and the other cognitive loads that are not specific to sustained visual attention.

## Footnotes

1 See for instance Grau et al. (2014) or Rao et al. (2014) for promising brain-to-brain communication interfaces.

2 See e.g. http://dreem.com for an example of BCI for the general public aimed at improving the quality of sleep.

3 The number of electromyographic artifacts made the recording unexploitable.
